# Insufficient dietary intake and symptom burden are associated with early nutritional decline in hospitalized patients with cancer

**DOI:** 10.1007/s00520-026-11410-6

**Published:** 2026-10-23

**Authors:** Camilla Horn Soares, Larissa Farinha Maffini, Heloisa Jacques Friedrich, Giovanna Strapasson Faccin, Thais Steemburgo

**Affiliations:** 1 Graduate Program in Food, Nutrition, and Health, Universidade Federal Do Rio Grande Do Sul, Porto Alegre, Rio Grande Do Sul, Brazil; 2 Hospital de Clínicas de Porto Alegre, Porto Alegre, Rio Grande Do Sul, Brazil; 3 Graduate Program in Medical Sciences, Endocrinology, Universidade Federal Do Rio Grande Do Sul, Porto Alegre, RS Brazil; 4 Department of Nutrition, Universidade Federal Do Rio Grande Do Sul, Porto Alegre, Rio Grande Do Sul, Brazil

**Keywords:** Food Intake, Neoplasms, Appetite, Anthropometry, Skeletal muscle, Muscle Strength

## Abstract

**Purpose:**

To assess energy and protein intake relative to nutritional prescriptions in hospitalized patients with cancer and examine associations with appetite, nutrition-related symptoms, cancer-related factors, and changes in body weight (BW), anthropometric, and muscle strength indicators.

**Methods:**

This longitudinal study included patients with cancer following during the first six days of hospitalization. Appetite and dietary intake were assessed on days 2, 4, and 6 using a Visual Analogue Scale (VAS) and Food Intake Visual Scale (FIVS), respectively. Reduced appetite was defined as mean VAS ≤ 70 mm and reduced dietary intake as mean FIVS < 75%. BW, calf circumference (CC), mid-upper arm circumference (MUAC), and handgrip strength (HGS) were assessed. Differences between prescribed and estimated energy and protein intake, associations, and longitudinal changes were analyzed.

**Results:**

Seventy-seven patients (62.6 ± 12.9 years; 54.5% male) were included, mostly with gastrointestinal cancer and advanced-stage disease. Reduced CC, MUAC, and HGS were observed in 83.1%, 53.2%, and 41.6%, respectively. Estimated energy and protein intake was significantly lower than prescribed amounts on all assessment days, with median deficits of 867–1012 kcal/day and 35.2–50.1 g/day, respectively. Reduced dietary intake was associated with anorexia, dry mouth, taste and smell alterations, and recent chemotherapy. Among men with reduced dietary intake, BW and MUAC decreased from day 2 to day 6.

**Conclusions:**

Energy and protein intake were consistently lower than prescribed amounts. Reduced dietary intake was associated with nutrition-related symptoms and cancer-related factors and with early declines in BW and MUAC among men.

**Supplementary Information:**

The online version contains supplementary material available at https://doi.org/10.1007/s00520-026-11410-6.

## Introduction

Malnutrition, a common condition among hospitalized patients with cancer, is influenced by multiple factors [1–3]. Patients with gastrointestinal, head and neck, and lung cancers are at high risk of malnutrition due to the combined effects of disease progression and the host’s response to the tumor [1, 4]. In gastrointestinal cancers, tumor-induced mechanical obstruction may further compromise food intake [1, 4]. In these patients, reduced dietary intake frequently arises from nutrition impact symptoms driven by both the local effects of the tumor and the adverse effects of anticancer therapies [5]. At the same time, tumor-induced systemic inflammation drives a hypercatabolic state characterized by accelerated breakdown of skeletal muscle proteins, which may occur with or without depletion of fat stores [5].

Overall, the association of malnutrition and reduced muscle mass with impaired response to cancer treatment, reduced quality of life, and poorer survival underscores the importance of their early detection and timely nutritional intervention to improve clinical outcomes [6, 7]. The European Society for Parenteral and Enteral Nutrition (ESPEN) guidelines for clinical nutrition in oncology recommend an energy intake of 25–30 kcal/kg/day and a protein intake of 1.0–1.5 g/kg/day [8]. Higher protein intakes (> 1.4 g/kg/day) have also been suggested to support the preservation of skeletal muscle mass [9]. Also, in older adults with cancer, higher protein and energy intakes may be required due to anabolic resistance, and nutritional supplementation is often necessary to help meet these increased demands [10].

Adequate energy and protein intake are essential to meet the increased and often fluctuating metabolic demands imposed by cancer and its treatment [10, 11]. However, oral intake is frequently compromised, primarily due to anorexia and other nutrition impact symptoms [2]. During hospitalization, reduced dietary intake is highly prevalent across clinical conditions, including cancer, and can lead to significant nutritional deficits within few days [12–16]. In this perspective, systematic assessment of dietary intake, from hospital admission through follow-up, is crucial to accurately quantify consumption, identifying inadequacies early, and guide adjustments in nutritional therapy [11, 17].

Although inadequate dietary intake is frequent among patients with cancer, evidence specifically addressing energy and protein intake during hospitalization remains limited and heterogeneous [13]. In hospitalized oncology patients, inadequate energy and protein intake have been documented, particularly among those at nutritional risk; however, previous investigations have focused primarily on associations with hospital outcomes, such as length of stay and readmission [13]. In patients hospitalized with colorectal cancer, reduced dietary intake has been associated with loss of appetite and other nutrition impact symptoms, based on qualitative assessment rather than quantitative measurement of energy and protein intake [12]. Similarly, a large multicenter study of hospitalized patients with cancer demonstrated strong associations between nutrition impact symptoms and malnutrition, particularly among patients with lung, upper gastrointestinal, and head and neck cancers, but did not quantify energy and protein intake [18]. In patients with head and neck and esophageal cancers, longitudinal data have further shown that prescribed energy and protein targets are frequently not achieved during cancer treatment, while these assessments have focused on the broader treatment trajectory rather than specifically on the hospitalization period [19].

Despite these findings, the interrelationships between inadequate dietary intake and nutrition impact symptoms during hospitalization remain insufficiently characterized. In particular, evidence regarding the adequacy of energy and protein intake relative to prescribed nutritional regimens throughout hospitalization in patients with gastrointestinal, lung, and head and neck cancers remains limited, especially in relation to appetite, symptom burden, cancer-related clinical factors, and early changes in body weight and muscle-related indicators. Therefore, the present study aimed to assess energy and protein intake in relation to nutritional prescriptions in these patients and to investigate their associations with appetite, nutrition impact symptoms, and cancer-related clinical factors. Additionally, we evaluated the relationship between insufficient intake and short-term changes in body weight and indicators of muscle mass and strength.

## Methods

### Study design and participants

This longitudinal study was conducted among patients with cancer admitted to the Hospital de Clínicas de Porto Alegre and followed for 6 days of hospitalization. The follow-up period was defined based on the median hospital stay reported in a previously published cohort study conducted by our research group [20]. Inclusion criteria were age ≥ 18 years, a diagnosis of gastrointestinal, lung, or head and neck cancer, and prescription of an exclusively oral diet, with or without oral nutritional supplementation. Patients admitted to the emergency department or intensive care unit, with reduced level of consciousness, prescribed no oral intake, receiving enteral or parenteral nutrition, presenting edema, or diagnosed with COVID-19 or multidrug-resistant infections were excluded. The study was approved by the Research Ethics Committee of the Hospital de Clínicas de Porto Alegre (protocol #2022.0432). Eligible patients were approached at the bedside by a trained researcher, who explained the study procedures and invited them to participate. Patients who agreed to participate provided written informed consent before data collection.

### Data collection

**Fig. 1:**
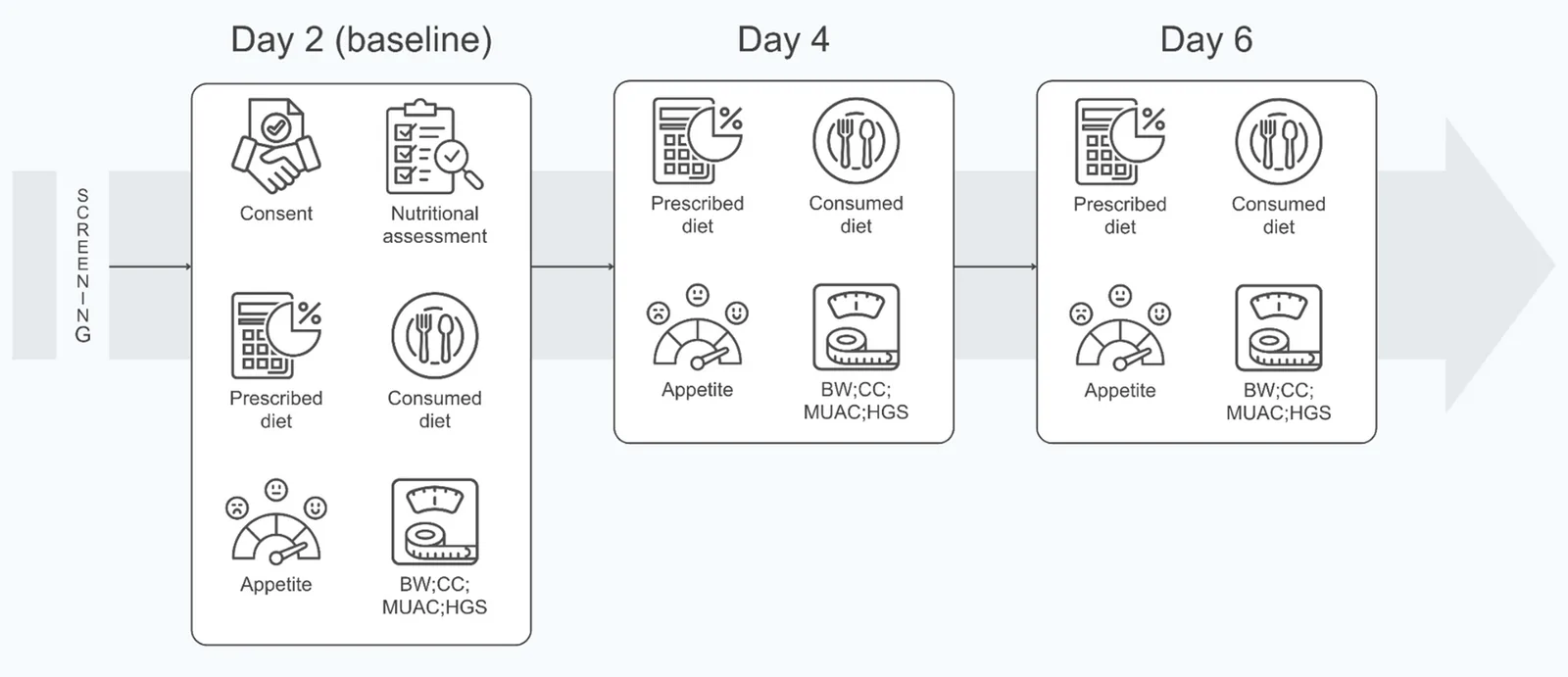
Flow diagram of the study. Abbreviations: BW = body weight; CC = calf circumference; MUAC = mid-upper arm circumference; HGS = hand grip strength

The flow diagram of the study is described in Fig. 1. Patient data were collected at the bedside by trained researchers at three points during hospitalization: day 2 (baseline), day 4, and day 6. Bedside assessments included nutritional assessment, dietary intake, appetite, body weight, calf circumference, mid-upper arm circumference, and handgrip strength.

### General data

Data was collected at the patients’ bedside within the first 48 h of hospital admission by a trained researcher. Sociodemographic characteristics, including age, sex, and education, were obtained from electronic medical records. Ethnicity was self-declared by the patient or a family member at hospital admission and subsequently retrieved from the electronic medical record. Smoking status (yes/no) and alcohol consumption (yes/no) were self-reported by the patients. Physical activity was self-reported by patients through the question: "Do you practice any kind of physical activity?" (yes/no), if yes, what activity, how many times a week do you practice and for how long. Clinical characteristics, including comorbidities, primary tumor site, disease stage, presence of metastases, and oncological treatment, were obtained from electronic medical records.

### Anthropometry

#### Body weight and body mass index

Body weight (BW) was measured on days 2, 4, and 6 of hospitalization using a calibrated digital hospital scale, under standardized conditions, with participants wearing light clothing and no shoes. Height (m) was measured once using a wall-mounted stadiometer. Body mass index (BMI) was calculated as weight (kg)/height (m^2^) and classified according to the World Health Organization (WHO) criteria [21].

### Malnutrition risk and malnutrition assessment

Nutritional risk and nutritional status were assessed by the research team on hospitalization day 2 (baseline), independently of routine clinical care assessments. Nutritional risk was screened using the Patient-Generated Subjective Global Assessment Short Form (PG-SGA SF), which evaluates weight history, food intake, nutrition impact symptoms, and functional capacity [22]. A score ≥ 4 was considered indicative of nutritional risk [22]. Nutritional status was assessed using the full Patient-Generated Subjective Global Assessment (PG-SGA), which combines patient-reported information on weight history, food intake, nutrition impact symptoms, and functional capacity with a professional component that considers diagnosis, age, metabolic stress, and physical examination [23]. Patients were classified as well as nourished (A), moderately or suspected of being malnourished (B), or severely malnourished (C) [23]. The PG-SGA was specifically developed for patients with cancer and captures nutrition impact symptoms that may directly affect dietary intake. Accordingly, these symptoms were investigated for their associations with energy and protein intake in the present study [22, 23].

### Muscle mass and muscle strength assessment

Calf circumference (CC), mid-upper arm circumference (MUAC), and hand grip strength (HGS), as indicators of muscle mass and strength, were assessed on hospitalization days 2, 4, and 6. CC was measured at the widest point of the calf using a flexible, non-elastic anthropometric tape (Cescorf®, Brazil). CC values ≤ 34 cm for men and ≤ 33 cm for women were considered indicative of low muscle mass, with adjustments according to BMI to minimize the effect of adiposity. These cutoffs were derived from the NHANES 1999–2006 population [24]. MUAC was measured with the participant seated, on the dominant arm, at the midpoint between the acromion and olecranon processes, using a non-elastic, flexible measuring tape (Cescorf®, Brazil). Reduced values were defined as < 28 cm for men and < 25 cm for women, with BMI-based adjustments; these cutoffs were also derived from the NHANES 1999–2006 population [25]. HGS was used as an indicator of muscle strength and was measured using a calibrated Jamar® hand dynamometer. Measurements were performed in the seated position, on the dominant hand, with the elbow flexed at 90°. Three attempts were recorded, and the highest value was considered maximal muscle strength. Muscle strength was classified as low using HGS cutoffs of < 27 kg for men and < 16 kg for women, based on the revised European consensus on sarcopenia (EWGSOP2) [26]. None of these cutoffs were specifically developed or validated in patients with cancer.

### Assessment of appetite and dietary intake

Appetite was assessed on hospitalization days 2, 4, and 6 using a 100-mm visual analogue scale (VAS), anchored from “no appetite at all” (0 mm) to “a very good appetite” (100 mm) [27]. To provide an overall measure of appetite, the mean VAS score across the three assessment days was calculated. Reduced appetite was defined using a cutoff of ≤ 70 mm [27].

Dietary intake was assessed at the same time points using the Food Intake Visual Scale (FIVS). Patients reported the proportion of hospital meals consumed during the preceding 24 h using the FIVS categories of almost all (100%), half (50%), little (25%), or nothing (0%) [16] (Supplementary Material 1). The mean percentage of dietary intake assessed by the FIVS across the three assessment days was calculated, and values < 75% were classified as reduced dietary intake [4, 8].

### Energy and protein prescription and estimated intake

Diet prescription data were obtained from electronic medical records on hospitalization days 2, 4, and 6, including prescribed energy and protein content. Energy and protein requirements were individually determined by the hospital clinical dietitian following nutritional assessment, based on nutrition therapy recommendations for patients with cancer (25–30 kcal/kg current body weight/day and > 1.0 g protein/kg current body weight/day) [8]. Prescribed and consumed energy and protein were expressed both as absolute values (kcal/day and g/day, respectively) and relative to current body weight (kcal/kg/day and g/kg/day, respectively).

Energy and protein intake were estimated based on the proportion of hospital meals consumed over the previous 24 h, as assessed by the research team using the FIVS [16]. The proportion consumed was applied to the energy and protein content of the corresponding meals to estimate actual intake, assuming that the percentage of food consumed reflected the same percentage of the prescribed energy and protein content. These estimates were calculated on hospitalization days 2, 4, and 6 and expressed as percentages of the prescribed amounts.

### Statistical analysis

The sample size was calculated to detect a difference of 9.32 g/day between prescribed and consumed protein, based on the difference reported in a previously published study [28]. Assuming a pooled standard deviation of 24.87 g, a statistical power of 90%, and a significance level of 5%, the required sample size was estimated at 77 patients. The calculation was performed using the PSS Health online tool.

The Kolmogorov–Smirnov test was used to assess data normality. Continuous variables were presented as mean (standard deviation) or median (interquartile range [IQR]), whereas categorical variables were presented as counts and percentages. Differences between prescribed diet and dietary intake were analyzed using the Wilcoxon signed-rank test.

Pearson’s chi-square test was used to assess the associations of reduced appetite (mean VAS ≤ 70 mm across hospitalization days 2, 4, and 6) and nutrition impact symptoms with reduced dietary intake (mean FIVS < 75% across the same assessment days). Pearson’s chi-square test was also used to assess the associations of cancer diagnosis, stage, and treatment with reduced appetite and reduced dietary intake. For cancer diagnosis, when a significant association was identified, adjusted residuals were examined to determine which categories contributed to the observed association. Longitudinal changes in nutritional indicators (BW, CC, MUAC, and HGS) according to dietary intake were analyzed using Generalized Estimating Equations for longitudinal data, stratified by sex.

Data analysis was performed using IBM SPSS Statistics version 25.0 (Chicago, IL, USA), and p-values < 0.05 were considered significant.

## Results

### General and clinical characteristics

A total of 77 patients (54.5% male) were included in the study. The mean age was 62.6 ± 12.9 years, and 63.6% were older adults. Baseline characteristics are summarized in Table 1. Gastrointestinal cancer was the most frequent cancer diagnosis (70.1%). Most patients had advanced-stage disease (stage III/IV; 84.6%), and 37.7% had metastasis. Regarding treatment, 54.5% received combined therapy, 11.7% underwent surgery alone, and 24.7% had not yet initiated treatment due to a recent diagnosis. Hypertension (53.2%) and type 2 diabetes (18.2%) were the most common comorbidities.

**Table 1:** Baseline general e clinical characteristics of 77 hospitalized patients with gastrointestinal, lung, and head and neck cancer

General	Values
Age (years)	62.6 ± 12.9
Older adults (≥ 60 years)	49 (63.6%)
Sex (male)	42 (54.5%)
Ethnicity (white)	60 (77.9%)
Education (≤ 8 years)	47 (61.0%)
Smoking (yes)	50 (64.9%)
Alcohol consumption (yes)	17 (22.1%)
Physical inactivity (yes)	73 (94.8%)
Clinical	
Cancer diagnosis	
Gastrointestinal	54 (70.1%)
Lung	19 (24.7%)
Head and neck	4 (5.2%)
Cancer staging	
I/II	10 (15.4%)
III/IV	55 (84.6%)
Metastatic disease	29 (37.7%)
Cancer treatment history	
Surgery	9 (11.7%)
Chemotherapy	4 (5.2%)
Combined treatment*	42 (54.5%)
Other	3 (3.9%)
No treatment to date	19 (24.7%)
Chronic diseases	
Hypertension	41 (53.2%)
Type 2 diabetes	14 (18.2%)
Dyslipidemia	6 (7.8%)

Data are expressed as n (%), mean ± standard deviation, and median (interquartile range)

^*^Combined treatment: more than one cancer treatment modality

### Nutritional characteristics

Table 2 describes the baseline nutritional characteristics of hospitalized patients. The median BW was 61.5 kg (IQR, 53.1–73.0), and the mean BMI was 23.5 ± 5.2 kg/m^2^. According to the PG-SGA SF, 84.4% of patients were at nutritional risk. Based on the PG-SGA, 80.6% were classified as moderately or severely malnourished (categories B or C). Regarding anthropometric indicators related to muscle mass, 58.4% of patients had low CC without BMI adjustment, whereas 83.1% had low CC after adjustment for BMI. Low MUAC was observed in 53.2% of patients without BMI adjustment and in 66.2% after adjustment for BMI. Regarding muscle strength, low HGS was identified in 41.6% of patients.

**Table 2:** Baseline nutritional characteristics of 77 hospitalized patients with gastrointestinal, lung, and head and neck cancer

Weigth and BMI	Values
Body weight (kg)	61.5 (53.1–73.0)
BMI (kg/m^2^)	23.5 ± 5.2
Underweight	26 (33.8%)
Normal weight	28 (36.4%)
Overweight/Obesity	23 (29.9%)
Malnutrition risk and malnutrition	
PG-SGA SF (score ≥ 4)	65 (84.4%)
PG-SGA	
Moderately or severely malnourished (B or C)	62 (80.6%)
Muscle mass assessment	
CC*	
Low CC _no adjusted for BMI_	45 (58.4%)
Low CC _adjusted for BMI_	64 (83.1%)
MUAC**	
Low MUAC _no adjusted for BMI_	41 (53.2%)
Low MUAC _adjusted for BMI_	51 (66.2%)
Muscle strength evaluation	
Low HGS***	32 (41.6%)

Data are expressed as n (%), mean ± standard deviation, and median (interquartile range)

*BMI* Body Mass Index, *PG-SGA SF* Patient Generated Subjective Global Assessment Short Form, *PG-SGA* Patient Generated Subjective Global Assessment, *CC* Calf Circumference, *MUAC* Mid-Upper Arm Circumference, *HGS* Hand Grip Strength

^*^Low CC: Male (≤ 34 cm); Female (≤ 33 cm); CC values were adjusted by patient’s BMI, in order to help to remove the confounding effects of adiposity [24]

^**^Low MUAC: Male (< 28 cm); Female (25 cm); MUAC values were adjusted by patient’s BMI, in order to help to remove the confounding effects of adiposity [25]

^***^Low HGS: Male (< 27 kg); Female (< 16 kg) [26]

### Nutrition impact symptoms and dietary intake

**Fig. 2:**
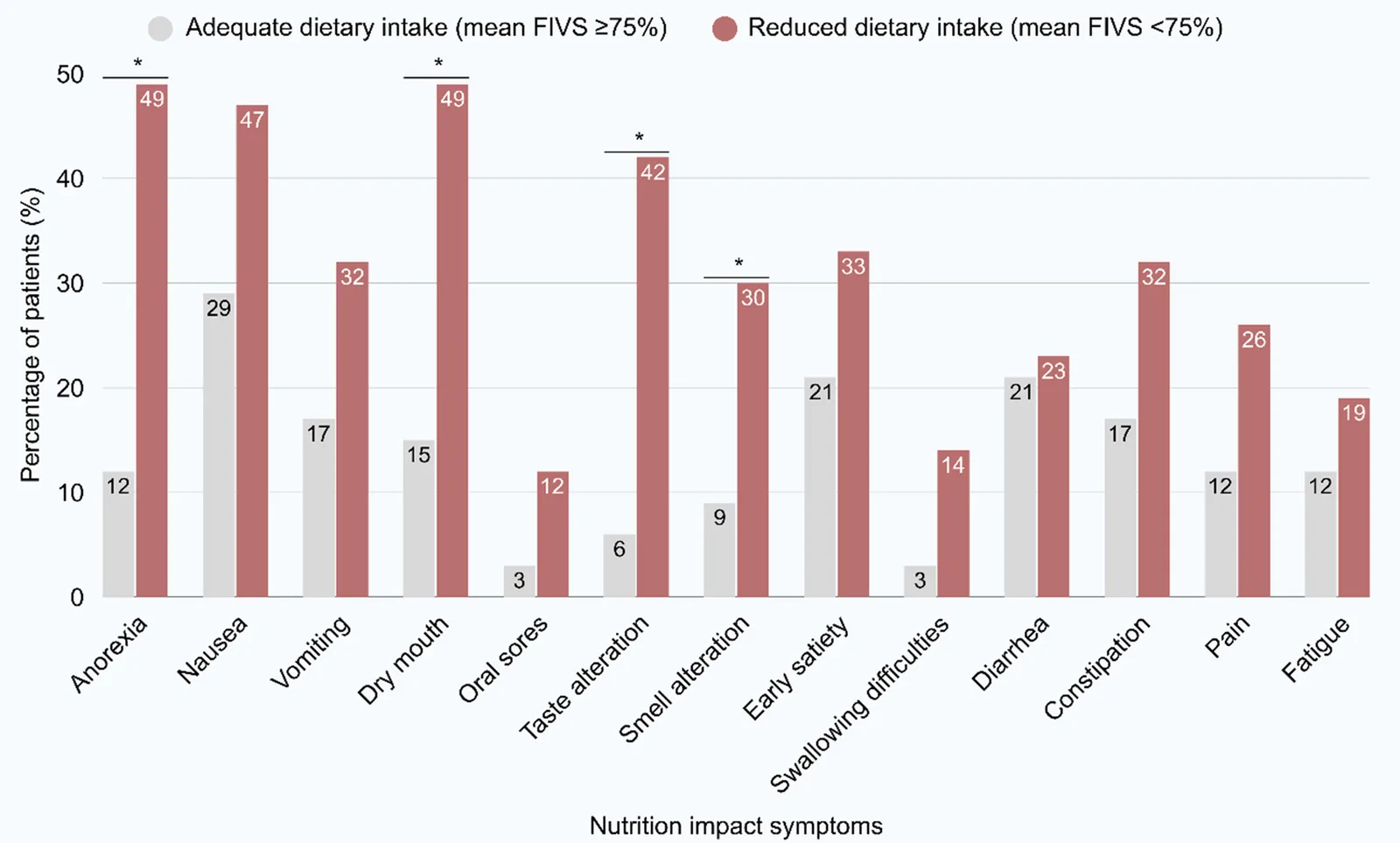
Relation between nutrition impact symptoms and dietary intake classified by mean FIVS across the three assessment days (*n = *77). *Indicates a statistically significant association

Figure 2 shows the prevalence of nutrition impact symptoms according to dietary intake, classified based on the mean FIVS across the three assessment days (< 75% and ≥ 75%). Patients with reduced dietary intake (mean FIVS < 75%) had a higher prevalence of anorexia (*p* = 0.001), dry mouth (*p* = 0.044), taste alteration (*p* = 0.001), and smell alteration (*p* = 0.044) than those with adequate dietary intake (mean FIVS ≥ 75%). In addition, dietary intake was strongly associated with appetite status: patients with reduced dietary intake predominantly had reduced appetite (88.4%), whereas those with adequate dietary intake mainly had good appetite (76.5%) (*p* < 0.001) (data not shown).

### Energy and protein prescription and estimated intake

Table 3 presents prescribed and estimated energy and protein intake on hospitalization days 2, 4, and 6. On all three assessment days, estimated energy and protein intake was significantly lower than the prescribed amounts, with median energy deficits ranging from 867 to 1012 kcal/day and median protein deficits from 35.2 to 50.1 g/day (p < 0.001 for all comparisons). Similar findings were observed when energy and protein were expressed relative to body weight, with median differences of up to 14.2 kcal/kg/day for energy and 0.75 g/kg/day for protein.

**Table 3:** Energy and protein prescription and estimated intake on hospitalization days 2, 4, and 6 (*n = *77)

Energy and protein	Hospitalization time		
	Day 2 (Baseline)	Day 4	Day 6
Energy (kcal/day)			
Prescribed	2007.8 (1829.7–2287.4)	2196.0 (2025.2–2519.2)	2161.8 (1950.6–2537.6)
Consumed	1269.7 (907.7–1996.6)	1387.1 (1037.3–2105.4)	1184.9 (876.2–1974.5)
Median of the differences	867.3 (0–1203.3)	932.0 (0–1241.8)	1012.3 (0–1333.7)
*p*-value^*^	< 0.001	< 0.001	< 0.001
Energy (kcal/kg/day)			
Prescribed	33.7 (28.6–40.8)	36.5 (29.8–43.1)	36.4 (29.2–43.9)
Consumed	20.7 (11.7–32.9)	24.9 (15.8–33.4)	19.9 (12.7–31.6)
Median of the differences	10.7 (0–20.3)	13.7 (0–21.6)	14.1 (0–24)
*p*-value^*^	< 0.001	< 0.001	< 0.001
Proteins (g/day)			
Prescribed	97.9 (87.3–110.7)	108.7 (97.1–117.7)	109.2 (93.4–120.2)
Consumed	55.4 (38.7–96.4)	59.1 (49.1–101.1)	57.4 (39.8–92.6)
Median of the differences	35.2 (0–55.3)	42.7 (0–58.3)	50.1 (0–65.9)
*p*-value^*^	< 0.001	< 0.001	< 0.001
Proteins (g/kg/dia)			
Prescribed	1.6 (1.37–1.94)	1.7 (1.5–2.1)	1.7 (1.5–2.1)
Consumed	1.0 (0.6–1.6)	1.1 (0.8–1.6)	0.9 (0.6–1.5)
Median of the differences	0.4 (0–1.0)	0.6 (0–1.0)	0.8 (0–1.2)
*p*-value^*^	< 0.001	< 0.001	< 0,001

^*^Wilcoxon test

### Cancer factors associated with appetite status and dietary intake

Table 4 presents cancer characteristics according to appetite status and dietary intake, classified based on the mean VAS and FIVS across the three assessment days. Cancer diagnosis was associated with reduced appetite and reduced dietary intake (p = 0.021 and p = 0.035, respectively). Although head and neck cancer showed the highest frequency of reduced appetite, adjusted residual analysis indicated that this tumor site did not significantly contribute to the observed association. In contrast, lung cancer showed a positive contribution, whereas gastrointestinal cancer showed a negative contribution. A similar pattern was observed for reduced dietary intake. Cancer stage was not associated with reduced appetite or reduced dietary intake. Chemotherapy in the previous three months was associated with reduced dietary intake (p = 0.046), but not with reduced appetite. Surgery and radiotherapy were not associated with either outcome.

**Table 4:** Cancer diagnosis, stage, and treatment according to reduced appetite (mean VAS ≤ 70 mm) and reduced dietary intake (mean FIVS < 75%) across the three assessment days (*n = *77)

	Appetite (VAS ≤ 70 mm)	*p*-value*	Dietary intake (FIVS < 75%)	*p*-value*
Cancer diagnosis				
Gastrointestinal	27 (50%) ^−2.7^		25 (46.3%)^−2.6^	
Lung	15 (78.9%)^2.0^	0.021	15 (78.9%)^2.3^	0.035
Head and Neck	4 (100%)^1.7^		3 (75%)^0.8^	
Cancer staging				
I	1 (33.3%)		1 (33.3%)	
II	5 (71.4%)	0.701	6 (85.7%)	0.364
III	17 (65.4%)		14 (53.8%)	
IV	18 (62.1%)		16 (55.2%)	
Cancer treatment (< 3 months)				
Surgery				
No	40 (60.6%)	0.704	37 (56.1%)	0.925
Yes	6 (54.5%)		6 (54.5%)	
Chemotherapy				
No	25 (53.2%)	0.142	22 (46.8%)	0.046
Yes	21 (70.0%)		21 (70.0%)	
Radiotherapy				
No	39 (56.5%)	0.091	36 (52.2%)	0.057
Yes	7 (87.5%)		7 (87.5%)	

^*^Chi-square test

Superscript values represent adjusted residuals from the chi-square test. Absolute values ≥ 1.96 indicate a statistically significant contribution to the association

(*p* < 0.05). Positive residuals indicate observed frequencies higher than expected, whereas negative residuals indicate observed frequencies lower than expected

### Changes in body weight, anthropometric and strength indicators according to dietary intake during hospitalization

Table 5 presents changes in body weight, anthropometric, and strength indicators according to dietary intake, classified based on the mean FIVS across the three assessment days. Among men, body weight showed a significant effect of time (p = 0.021) and a significant time-by-dietary intake interaction (p = 0.002). Patients with reduced dietary intake (mean FIVS < 75%) exhibited a progressive decrease in body weight from day 2 to day 6, with the estimated mean decreasing by 1.02 kg (from 66.25 to 65.23 kg), whereas body weight remained stable among those with adequate dietary intake (mean FIVS ≥ 75%). For MUAC adjusted for BMI, a significant time-by-dietary intake interaction was also observed among men (p = 0.008). MUAC decreased over time in patients with reduced dietary intake, with the estimated mean decreasing by 0.21 cm (from 24.45 to 24.24 cm) between days 2 and 6, while remaining stable in those with adequate dietary intake. Among women, no significant effects of time, dietary intake group, or time-by-dietary intake interaction were observed for any of the evaluated indicators.

**Table 5:** Longitudinal changes in nutritional indicators in hospitalized patients with cancer according to dietary intake classified by mean FIVS across the three assessment days (*n = *77)

Variable	Hospitalization time (days)	Reduced dietary intake (FIVS < 75%) Mean (SE)	Adequate dietary intake (FIVS ≥ 75%) Mean (SE)	p-value*		
				Time	Group	Interaction
Body weight (kg)						
Men	2	66.25 (3.03)	66.19 (4.00)	0.021	0.936	0.002
	4	65.70 (3.02)	65.83 (3.92)			
	6	65.23 (3.04)	66.36 (3.93)			
Women	2	60.69 (2.76)	60.63 (3.94)	0.059	0.856	0.093
	4	58.70 (2.70)	60.53 (3.83)			
	6	59.82 (2.68)	60.60 (3.88)			
CC _adjusted for BMI_ (cm)						
Men	2	31.18 (0.62)	32.19 (0.62)	0.157	0.119	0.064
	4	30.80 (0.64)	32.23 (0.62)			
	6	30.61 (0.61)	32.24 (0.60)			
Women	2	30.24 (0.52)	32.19 (0.87)	0.173	0.071	0.706
	4	30.22 (0.53)	32.07 (0.86)			
	6	30.10 (0.58)	31.84 (0.76)			
MUAC _adjusted for BMI_ (cm)						
Men	2	24.45 (0.63)	24.81 (0.65)	0.264	0.422	0.008
	4	24.32 (0.63)	25.26 (0.63)			
	6	24.24 (0.68)	25.10 (0.59)			
Women	2	24.55 (0.65)	26.30 (0.89)	0.834	0.108	0.453
	4	24.54 (0.63)	26.12 (0.91)			
	6	24.40 (0.69)	26.40 (0.91)			
HGS (kg)						
Men	2	26.95 (1.67)	28.30 (2.10)	0.918	0.637	0.888
	4	27.36 (1.58)	28.40 (2.54)			
	6	26.91 (1.54)	28.50 (2.53)			
Women	2	15.86 (1.52)	19.71 (1.67)	0.754	0.147	0.183
	4	16.29 (1.53)	19.57 (1.72)			
	6	16.57 (1.28)	18.36 (1.37)			

Data is expressed as mean (standard error). ^*^Generalized Estimating Equations (GEE) test

*BMI* Body Mass Index, *CC* Calf Circumference, *MUAC* Mid-Upper Arm Circumference, *HGS* Hand Grip Strength

## Discussion

In this longitudinal study of hospitalized patients with cancer, estimated energy and protein intake was consistently below prescribed amounts on hospitalization days 2, 4, and 6. Reduced dietary intake was associated with nutrition impact symptoms, particularly anorexia, xerostomia, and taste or smell alterations. Tumor site was associated with both reduced appetite and reduced dietary intake, whereas recent chemotherapy was associated only with reduced dietary intake, highlighting the influence of disease- and treatment-related factors. Importantly, reduced dietary intake was accompanied by declines in BW and MUAC among men from day 2 to day 6, emphasizing the importance of early identification and close nutritional monitoring in oncological care settings.

Baseline nutritional assessment showed a markedly compromised nutritional status, with 84.4% of patients identified as being at nutritional risk according to the PG-SGA SF and 80.6% classified as malnourished by the PG-SGA. These findings are consistent with the high nutritional vulnerability previously reported in patients with gastrointestinal, head and neck, and lung cancers [1, 3]. The high prevalence observed may partly reflect the ability of these cancer-specific instruments to capture nutrition impact symptoms and functional impairment [22, 23], factors that are particularly relevant in the context of dietary intake. Importantly, these findings highlight the nutritional vulnerability of this population at hospital admission and provide the clinical context for the insufficient energy and protein intake and early nutritional changes observed during hospitalization. In addition, a high prevalence of low anthropometric indicators potentially reflecting reduced muscle reserves was observed, based on CC (58.4%) and MUAC (53.2%), increasing further with BMI-adjusted criteria. Although these measures do not directly assess skeletal muscle mass, low CC and MUAC have been associated with adverse clinical and nutritional outcomes in cancer populations, including patients with diagnoses at high nutritional risk [29–31]. Low muscle strength by HGS was observed in 41.6% of patients, consistent with previous findings and is clinically relevant as it may predict chemotherapy toxicity and adverse outcomes [20, 32]. Reduced HGS was also associated with higher mortality, poorer functional status, and decreased physical performance, particularly in patients with advanced-stage cancer [33]. Notably, in our study, most patients had advanced-stage cancer, which may contribute to the considerable occurrence of functional muscle impairment in this population.

Nutrition impact symptoms were associated with reduced dietary intake, consistent with previous studies [12, 15, 34]. In our sample, anorexia, dry mouth, and taste and smell alterations were significantly associated with reduced dietary intake (FIVS < 75%). Although the adverse effects of these symptoms are well established in patients with cancer [15, 18, 34], our findings extend previous evidence by showing their association with reduced dietary intake, defined as a mean FIVS < 75% across dietary assessments conducted on hospitalization days 2, 4, and 6. This is clinically relevant because hospitalized patients with cancer may already present a high nutritional vulnerability, and persistent symptoms may further limit their ability to meet nutritional requirements during this critical period. Anorexia has a multifactorial etiology involving treatment effects and inflammation-related neuroendocrine dysregulation, which may suppress appetite and compromise energy and protein intake [1, 2]. Xerostomia, frequently associated with chemotherapy- and radiotherapy-induced salivary gland dysfunction [35], may also contribute to reduced dietary intake through its association with taste and smell alterations [36–38]. In our sample, recent exposure to chemotherapy and radiotherapy may have contributed to the occurrence of these symptoms. These findings reinforce the importance of identifying and managing nutrition impact symptoms during hospitalization as potential barriers to adequate dietary intake.

In our study, only 44.2% of patients achieved a mean FIVS ≥ 75% based on the three assessments conducted on hospitalization days 2, 4, and 6. Estimated energy and protein intake was significantly below prescribed amounts on all three assessment days, with median energy deficits ranging from 867 to 1012 kcal/day and median protein deficits from 35.2 to 50.1 g/day (p < 0.001 for all comparisons). These findings aligned with studies show that hospitalized oncology patients rarely meet nutritional requirements despite individualized prescriptions [13, 14]. Previous studies show that only 9.1% of hospitalized patients with cancer meet caloric goals, and just 25% achieve recommended protein intake [13]. Patients at nutritional risk consume significantly less energy and protein, highlighting their vulnerability during hospitalization [13]. This inadequacy is not limited to oral intake but also occurs in patients receiving enteral nutrition [14]. Energy deficits remain substantial and have been associated with increased hospital mortality [14]. Reduced dietary intake, combined with systemic inflammation, is a recognized determinant of malnutrition in patients with cancer [4]. In our study, a high proportion of patients were classified as malnourished (80.6%), based on assessments conducted within the first 48 h, reflecting their condition at admission. However, estimated energy and protein intake was consistently below prescribed amounts on hospitalization days 2, 4, and 6, indicating inadequate intake across the three assessment days that may further compromise nutritional status.

Although our study focused on cancer diagnoses linked to nutritional impairment, this variable remained significantly associated with appetite and dietary intake. Lung cancer showed a strong association with reduced appetite and intake during hospitalization. Other findings also report that reduced intake in lung cancer, while gastrointestinal tumors are not always associated with this outcome [15]. However, a meta-analysis indicates higher malnutrition prevalence in gastrointestinal cancers [3], possibly due to impairments in nutrient digestion and absorption [1, 4], despite the impact of lung cancer on dietary intake. Furthermore, chemotherapy within the previous three months was associated with reduced dietary intake, although not with appetite. This reduction may result from side effects such as nausea, diarrhea, and taste alterations, leading to lower consumption and energy deficits [39]. Taste disturbances can appear early during chemotherapy and persist for weeks or months, often following a cyclical pattern. These changes typically normalize within 3 to 6 months after treatment completion [40]. In our study, taste and smell alterations were associated with reduced dietary intake (mean FIVS < 75% across the three assessment days), and nearly 40% of patients had recently received chemotherapy, suggesting a potential contribution of treatment-related factors to reduced dietary intake.

To the best of our knowledge, this is the first longitudinal study to assess dietary intake and its association with changes in body weight, anthropometric measures, and muscle strength in hospitalized patients with cancer. Among men, body weight decreased from day 2 to day 6 in those classified as having reduced dietary intake, whereas it remained stable in those with adequate intake, suggesting early changes in nutritional status. Unintentional weight loss is often multifactorial and may reflect reduced dietary intake, nutrition impact symptoms, and cancer cachexia, contributing to its clinical relevance in this population [41, 42]. Moreover, systemic inflammation and cachexia-related metabolic alterations may interact with nutrition impact symptoms and reduced dietary intake, further contributing to weight loss and nutritional deterioration in patients with cancer [5]. Evidence shows that even moderate weight loss is associated with poorer survival, highlighting its prognostic importance in oncology [41]. A recent meta-analysis of 73 cohort studies showed that decreases in body weight are consistently associated with poorer survival across cancer diagnosis, highlighting its prognostic importance [42]. Similarly, in patients with lung cancer, even moderate weight loss (≥ 5% over 2 months) predicts reduced overall and progression-free survival [41]. These associations are independent of cancer diagnosis or stage [41].

Body weight changed significantly from day 2 to day 6, with a significant time-by-dietary intake interaction among men. In those classified as having reduced dietary intake based on the three assessment days, estimated mean body weight decreased by 1.02 kg, whereas it remained stable among those with adequate dietary intake. A significant reduction in MUAC was also observed in men with reduced dietary intake. These findings suggest that reduced dietary intake may be accompanied by early changes in body weight and peripheral anthropometric measures during hospitalization. However, because MUAC is an indirect anthropometric measure influenced by both muscle and subcutaneous tissue, these changes should not be interpreted as direct evidence of skeletal muscle loss. These findings are clinically relevant, as low MUAC has been associated with poorer quality of life and adverse outcomes in patients with cancer [30, 31, 43]. No significant changes were observed in CC, suggesting that this anthropometric measure may be less responsive to short-term nutritional changes. Similarly, HGS showed no longitudinal changes, indicating no detectable change in muscle strength over the observation period. In contrast, no significant longitudinal changes in body weight or anthropometric indicators were observed among women. The reasons for these sex-specific differences remain uncertain and may partly reflect the short observation period and reduced sample size after stratification by sex. Further studies with larger samples are needed to determine whether these differences reflect sex-specific patterns of nutritional decline during hospitalization.

## Importance of the findings for routine clinical practice

The present findings demonstrate that estimated energy and protein intake were lower than prescribed amounts on all three assessment days, highlighting the challenges of achieving nutritional requirements in hospitalized patients with cancer. Nutrition-related symptoms were strongly associated with reduced dietary intake, emphasizing the importance of their early recognition and management in routine clinical practice. In this context, the PG-SGA, in either its short or complete form, may be particularly useful for identifying symptom burden and supporting targeted nutritional interventions. Nutritional care should align with ESPEN recommendations, prioritizing individualized nutritional counseling, management of nutrition impact symptoms, and energy and protein-dense strategies, including oral nutritional supplements when indicated [8]. In addition, simple measures such as body weight and MUAC may help identify early nutritional decline during hospitalization and support timely adjustment of nutritional care.

### Limitations of the study

The present study had some limitations. Assessments were restricted to the first six days of hospitalization, capturing the early and clinically critical phase but not the entire hospital stay. Dietary intake was estimated using a validated hospital tool, although this approach does not provide information on the consumption of individual meal components and may not account for unrecorded nutritional supplementation [9, 10]. Additionally, estimating energy and protein intake based on the overall proportion of meal consumed assumes proportional consumption of all meal components. However, patients may selectively consume or leave specific foods with different energy and protein contents. Therefore, this approach may have resulted in some degree of over- or underestimation of actual energy and protein intake, which should be considered when interpreting the quantitative dietary intake estimates. We also acknowledge the smaller number of patients with head and neck cancer compared with the other cancer diagnoses, and this has been noted as a limitation when interpreting the findings for this subgroup.

Anthropometric measures and body weight may have been influenced by interobserver variability and fluid balance, despite standardized procedures. Furthermore, CC and MUAC are indirect anthropometric measures and do not provide direct measurements of skeletal muscle mass, while HGS reflects muscle strength rather than muscle mass. Therefore, changes in these measures should not be interpreted as direct evidence of changes in skeletal muscle mass. Nevertheless, their simplicity, low cost, and feasibility at the bedside support their use for nutritional and functional monitoring in routine clinical practice. Another limitation is that malnutrition was not diagnosed using the Global Leadership Initiative on Malnutrition (GLIM) framework, as this approach was not predefined in the original study protocol. Although CC and MUAC provided indirect anthropometric information related to muscle mass and HGS assessed muscle strength, these measures were collected as complementary indicators rather than as components of a standardized GLIM assessment. Therefore, the absence of a GLIM-based diagnosis may limit the integration of reduced muscle mass into the nutritional assessment and comparability with studies using GLIM criteria. Additionally, the focus on high-risk cancer diagnosis may limit generalizability, but it strengthens the clinical relevance of identifying factors associated with inadequate intake and early nutritional decline in a vulnerable population.

## Conclusion

In hospitalized patients with gastrointestinal, head and neck, and lung cancers, estimated energy and protein intake were lower than prescribed amounts on all three assessment days. Reduced dietary intake was associated with nutrition impact symptoms, cancer diagnosis, and recent chemotherapy, and was accompanied by early and clinically relevant declines in BW and MUAC during hospitalization. However, these findings should be interpreted within the multifactorial context of cancer-related nutritional decline. Cancer diagnosis also emerged as a key determinant of both reduced appetite and dietary intake. These findings emphasize that insufficient dietary intake is an early and clinically significant event in this population, warranting prompt recognition and intervention. Systematic monitoring of dietary intake and symptom burden, coupled with early, individualized nutritional strategies, is essential to prevent nutritional deterioration and improve clinical outcomes during hospitalization.

## Supplementary Information

Below is the link to the electronic supplementary material.

Supplementary file1 (DOCX 189 KB)

## Data Availability

No datasets were generated or analysed during the current study.

## References

[1] Arends J, Baracos V, Bertz H, Bozzetti F, Calder PC, Deutz NEP, Erickson N, Laviano A, Lisanti MP, Lobo DN, McMillan DC, Muscaritoli M, Ockenga J, Pirlich M, Strasser F, de van der Schueren M, Van Gossum A, Vaupel P, Weimann A (2017) ESPEN expert group recommendations for action against cancer-related malnutrition. Clin Nutr 36:1187–1196. 10.1016/j.clnu.2017.06.017 28689670

[2] Hosseini SM, Salari N, Darvishi N, Siahkamari Z, Rahmani A, Shohaimi S, Faghihi SH, Mohammadi M (2025) Prevalence of severe malnutrition in cancer patients: a systematic review and meta-analysis. J Health Popul Nutr 44:252. 10.1186/s41043-025-01006-x PMC12255005 40652267

[3] Zhang J, Quan Y, Wang X, Wei X, Shen X, Li X, Liang T (2025) Global epidemiological characteristics of malnutrition in cancer patients: a comprehensive meta-analysis and systematic review. BMC Cancer 25:1191. 10.1186/s12885-025-14558-2 PMC12275410 40684092

[4] Arends J, Bachmann P, Baracos V, Barthelemy N, Bertz H, Bozzetti F, Fearon K, Hütterer E, Isenring E, Kaasa S, Krznaric Z, Laird B, Larsson M, Laviano A, Mühlebach S, Muscaritoli M, Oldervoll L, Ravasco P, Solheim T, Strasser F, de van der Schueren M, Preiser JC (2017) ESPEN guidelines on nutrition in cancer patients. Clin Nutr 36:11–48. 10.1016/j.clnu.2016.07.015 27637832

[5] Arends J (2024) Malnutrition in cancer patients: causes, consequences and treatment options. Eur J Surg Oncol 50:107074. 10.1016/j.ejso.2023.107074 37783594

[6] Muscaritoli M, Arends J, Aapro M (2019) From guidelines to clinical practice: a roadmap for oncologists for nutrition therapy for cancer patients. Ther Adv Med Oncol 11:1758835919880084. 10.1177/1758835919880084 PMC6854759 31762796

[7] Crestani MS, Grassi T, Steemburgo T (2022) Methods of nutritional assessment and functional capacity in the identification of unfavorable clinical outcomes in hospitalized patients with cancer: a systematic review. Nutr Rev 80:786–811. 10.1093/nutrit/nuab090 34850196

[8] Muscaritoli M, Arends J, Bachmann P, Baracos V, Barthelemy N, Bertz H, Bozzetti F, Hütterer E, Isenring E, Kaasa S, Krznaric Z, Laird B, Larsson M, Laviano A, Mühlebach S, Oldervoll L, Ravasco P, Solheim TS, Strasser F, de van der Schueren M, Preiser JC, Bischoff SC (2021) ESPEN practical guideline: clinical nutrition in cancer. Clin Nutr 40:2898–2913. 10.1016/j.clnu.2021.02.005 33946039

[9] Capitão C, Coutinho D, Neves PM, Capelas ML, Pimenta NM, Santos T, Mäkitie A, Ravasco P (2022) Protein intake and muscle mass maintenance in patients with cancer types with high prevalence of sarcopenia: a systematic review. Support Care Cancer 30:3007–3015. 10.1007/s00520-021-06633-8 34697674

[10] Maffini LF, Soares CH, Beuren AG, Ferrari GT, Friedrich HJ, Camargo GB, Faccin GS, Gabrielli CP, Stefani GP, Oliveira VR, Steemburgo T (2025) An overview of the importance of muscle health in oncology: assessment, preservation, and nutritional recovery strategies. Curr Nutr Rep 14:130. 10.1007/s13668-025-00717-1 41460619

[11] Soares CH, Beuren AG, Friedrich HJ, Gabrielli CP, Stefani GP, Steemburgo T (2024) The importance of nutrition in cancer care: a narrative review. Curr Nutr Rep 13:950–965. 10.1007/s13668-024-00578-0 39278864

[12] van der Werf A, Arthey K, Hiesmayr M, Sulz I, Schindler K, Laviano A, Langius J, de van der Schueren M (2018) The determinants of reduced dietary intake in hospitalised colorectal cancer patients. Support Care Cancer 26:2039–2047. 10.1007/s00520-018-4044-1 PMC5919993 29352452

[13] Meure CM, Steer B, Porter J (2023) Nutritional intake, hospital readmissions and length of stay in hospitalised oncology patients. Cancers (Basel) 15:1488. 10.3390/cancers15051488 PMC10000899 36900278

[14] Gabrielli CP, Steemburgo T (2024) Adequate calorie and protein administration via enteral nutrition may contribute to improved 30-day survival in patients with solid tumors at nutritional risk. Clin Nutr ESPEN 59:279–286. 10.1016/j.clnesp.2023.12.014 38220387

[15] Shakhshir MH, Salameh HT, Amer R, Zyoud SH (2024) An evaluation of nutritional impact symptoms and their association with reduced dietary intake in patients with solid tumors at tertiary care hospitals: a multicenter, cross-sectional study from Palestine. BMC Cancer 24:524. 10.1186/s12885-024-12289-4 PMC11046763 38664802

[16] Hiesmayr M, Schindler K, Pernicka E, Schuh C, Schoeniger-Hekele A, Bauer P, Laviano A, Lovell AD, Mouhieddine M, Schuetz T, Schneider SM, Singer P, Pichard C, Howard P, Jonkers C, Grecu I, Ljungqvist O (2009) Decreased food intake is a risk factor for mortality in hospitalised patients: the NutritionDay survey 2006. Clin Nutr 28:484–491. 10.1016/j.clnu.2009.05.013 19573957

[17] Jin S, Cong M, Zhang L, Wang Y, Qin D, Lu Q (2020) Validation of a simple diet self-assessment tool (SDSAT) in head and neck cancer patients undergoing radiotherapy. Eur J Oncol Nurs 44:101702. 10.1016/j.ejon.2019.101702 31816506

[18] de Pinho NB, Martucci RB, Rodrigues VD, D’Almeida CA, Thuler LCS, Saunders C, Jager-Wittenaar H, Peres WAF (2019) Malnutrition associated with nutrition impact symptoms and localization of the disease: Results of a multicentric research on oncological nutrition. Clin Nutr 38(3):1274–1279. 10.1016/j.clnu.2018.05.010 29853223

[19] Martin L, Findlay M, Bauer JD, Dhaliwal R, de van der Schueren M, Laviano A, Widaman A, Baracos VE, Day AG, Gramlich LM (2022) A multi-site, international audit of malnutrition risk and energy and protein intakes in patients undergoing treatment for head neck and esophageal cancer: results from INFORM. Nutrients 14(24):5272. 10.3390/nu14245272 PMC9785112 36558428

[20] Crestani MS, Stefani GP, Scott LM, Steemburgo T (2023) Accuracy of the GLIM criteria and SGA compared to PG-SGA for the diagnosis of malnutrition and its impact on prolonged hospitalization: a prospective study in patients with cancer. Nutr Cancer 75:1177–1188. 10.1080/01635581.2023.2184748 36892543

[21] World Health Organization (2000) Obesity: preventing and managing the global epidemic: report of a WHO consultation. World Health Organization, Geneva 11234459

[22] Jager-Wittenaar H, Ottery FD (2017) Assessing nutritional status in cancer: role of the Patient-Generated Subjective Global Assessment. Curr Opin Clin Nutr Metab Care 20:322–329. 10.1097/MCO.0000000000000389 28562490

[23] Ottery FD (1996) Definition of standardized nutritional assessment and interventional pathways in oncology. Nutrition 12:S15–S19. 10.1016/0899-9007(96)90011-8 8850213

[24] Gonzalez MC, Mehrnezhad A, Razaviarab N, Barbosa-Silva TG, Heymsfield SB (2021) Calf circumference: cutoff values from the NHANES 1999–2006. Am J Clin Nutr 113:1679–1687. 10.1093/ajcn/nqab029 PMC8433492 33742191

[25] Costa-Pereira JP, Prado CM, Heymsfield SB, Fayh AP, Menezes-Júnior LA, Cabral PC, Diniz AS, Gonzalez MC (2025) Arm circumference as a marker of muscle mass: cutoff values from NHANES 1999–2006. Am J Clin Nutr 122:1809–1818. 10.1016/j.ajcnut.2025.09.034 41015191

[26] Cruz-Jentoft AJ, Bahat G, Bauer J, Boirie Y, Bruyère O, Cederholm T, Cooper C, Landi F, Rolland Y, Sayer AA, Schneider SM, Sieber CC, Topinkova E, Vandewoude M, Visser M, Zamboni M (2019) Sarcopenia: revised European consensus on definition and diagnosis. Age Ageing 48:16–31. 10.1093/ageing/afy169 PMC6322506 30312372

[27] Blauwhoff-Buskermolen S, Ruijgrok C, Ostelo RW, de Vet HCW, Verheul HMW, de van der Schueren MAE, Langius JAE (2016) The assessment of anorexia in patients with cancer: cut-off values for the FAACT-A/CS and the VAS for appetite. Support Care Cancer 24:661–666. 10.1007/s00520-015-2826-2 PMC4689771 26160463

[28] Simzari K, Vahabzadeh D, Nouri Saeidlou S, Khoshbin S, Bektas Y (2017) Food intake, plate waste and its association with malnutrition in hospitalized patients. Nutr Hosp 34:1376–1381 10.20960/nh.1102 29280654

[29] Scott LM, Stefani GP, Soares CH, Crestani MS, Steemburgo T (2024) Low calf circumference is associated with prolonged hospital stay in older patients with solid tumors: a secondary analysis of a cohort study. Nutr Cancer 76:726–735. 10.1080/01635581.2024.2364390 38909290

[30] Xie H, Wei L, Wang S, Shi H, Chen J (2025) Prognostic significance of anthropometric measurements in gastric cancer: a multicenter cohort study. Support Care Cancer 33:783. 10.1007/s00520-025-09849-0 40801979

[31] Maffini LF, Costa-Pereira JP, Soares CH, Beretta MV, Friedrich HJ, Stefani GP, Prado CM, Gonzalez MC, Steemburgo T (2026) Arm circumference as a predictor of cancer outcomes: validity of proposed cutoff points and BMI adjustment factors. Nutrition 151:113335. 10.1016/j.nut.2026.113335 42475886

[32] Papadopoulos E, Rozenberg D, Wong AKO, Law SHC, Costa S, Cheung AM, Alibhai SMH (2025) Skeletal muscle index, grip strength, and physical performance as predictors of severe chemotherapy toxicity among older adults with malignancy. PLoS ONE 20:e0336968. 10.1371/journal.pone.0336968 PMC12629486 41259322

[33] Hadzibegovic S, Porthun J, Lena A, Weinländer P, Lück LC, Potthoff SK, Rösnick L, Fröhlich AK, Ramer LV, Sonntag F, Wilkenshoff U, Ahn J, Keller U, Bullinger L, Mahabadi AA, Totzeck M, Rassaf T, von Haehling S, Coats AJS, Anker SD, Roeland EJ, Landmesser U, Anker MS (2023) Hand grip strength in patients with advanced cancer: a prospective study. J Cachexia Sarcopenia Muscle 14:1682–1694. 10.1002/jcsm.13248 PMC10401539 37318103

[34] de Pinho NB, Martucci RB, Rodrigues VD, D’Almeida CA, Thuler LCS, Saunders C, Jager-Wittenaar H, Peres WAF (2020) High prevalence of malnutrition and nutrition impact symptoms in older patients with cancer: Results of a Brazilian multicenter study. Cancer 126(1):156–164. 10.1002/cncr.32437 31497875

[35] Hosseini MS, Sanaie S, Mahmoodpoor A, Jabbari Beyrami S, Jabbari Beyrami H, Fattahi S, Jahanshahlou F, Zarei M, Rahimi Mamaghani A, Kuchaki Rafsanjani M (2024) Cancer treatment-related xerostomia: basics, therapeutics, and future perspectives. Eur J Med Res 29:571. 10.1186/s40001-024-02167-x PMC11607820 39614391

[36] Zhang B, Zhang N, Zhang Y, Yan J, Chen L, He H, Sun S, Zhang Y, Zhang M (2025) Prevalence and risk factors of chemotherapy-induced taste alterations among cancer patients: a systematic review and meta-analysis. Eur J Oncol Nurs 74:102735. 10.1016/j.ejon.2024.102735 39579474

[37] Pehlivan Köksal Z, Nural N (2025) The effect of taste alteration on malnutrition and quality of life in patients undergoing chemotherapy. Support Care Cancer 33:369. 10.1007/s00520-025-09431-8 PMC11985614 40210799

[38] Liang H, Yang H, Yin L, Wang L, Jiu E, Li B, Pang H (2025) Correlation between taste and smell alterations and quality of life in patients treated with radiotherapy for nasopharyngeal carcinoma: a cross-sectional study in China. Support Care Cancer 33:435. 10.1007/s00520-025-09484-9 PMC12041080 40299138

[39] de Freitas JS, de Oliveira Pedron ÉL, de Lima E Silva Aliprandi J, Laurentino GC, Gatti AL, Forones NM, de Cássia de Aquino R (2022) The effect of chemotherapy on dietary intake and nutritional status in patients with colorectal neoplasms and the importance of nutritional counseling. Support Care Cancer 30:3885–3891. 10.1007/s00520-022-06794-0 35043217

[40] Kiss N, Symons K, Hewitt J, Davis H, Ting C, Lee A, Boltong A, Tucker RM, Tan SY (2021) Taste function in adults undergoing cancer radiotherapy or chemotherapy, and implications for nutrition management: a systematic review. J Acad Nutr Diet 121:278–304. 10.1016/j.jand.2020.08.014 33071205

[41] Zhang J, Tang X, Zhang W, Xu Y, Zhang H, Fan Y (2025) Weight loss as a predictor of reduced survival in patients with lung cancer: a systematic review with meta-analysis. Int J Obes (Lond) 49:13–20. 10.1038/s41366-024-01642-z 39363034

[42] Wen H, Deng G, Shi X, Liu Z, Lin A, Cheng Q, Zhang J, Luo P (2024) Body mass index, weight change, and cancer prognosis: a meta-analysis and systematic review of 73 cohort studies. ESMO Open 9:102241. 10.1016/j.esmoop.2024.102241 PMC10925937 38442453

[43] Kim YJ, Hiratsuka Y, Suh SY, Won SH, Jung EH, Kang B, Lee SW, Ahn HY, Suh KJ, Kim JW, Kim SH, Kim JW, Lee KW, Kim JH, Lee JS (2022) Performance of mid-upper arm circumference and other prognostic indices based on inflammation and nutrition in oncology outpatients: a tertiary cancer center study. Ann Palliat Med 11:3171–3180. 10.21037/apm-22-481 36226642

