# Supplementary material for: Insufficient dietary intake and symptom burden are associated with early nutritional decline in hospitalized patients with cancer
Source: Support Care Cancer. 2026 Oct 23;34(11):1147. doi: 10.1007/s00520-026-11410-6 (PMC13694521; doi:10.1007/s00520-026-11410-6)
Supplement: Supplementary file 1 [file 520_2026_11410_MOESM1_ESM.docx]

**Supplementary Material 1**
